# Prevalence of multimorbidity and associated factors in the Brazilian working population

**DOI:** 10.47626/1679-4435-2020-568

**Published:** 2021-02-11

**Authors:** Ana Clara Dantas de Souza, Isabelle Ribeiro Barbosa, Dyego Leandro Bezerra de Souza

**Affiliations:** Universidade Federal do Rio Grande do Norte, Programa de Pós-Graduação em Saúde Coletiva - Natal (RN), Brazil

**Keywords:** multimorbidity, occupational health, absenteeism/presenteeism, occupational accident

## Abstract

**Introduction:**

According to the World Health Organization (2018), recent changes in the epidemiological profile of working populations point to an increase in non-communicable chronic illnesses and a decrease in communicable chronic illnesses.

**Objectives:**

To estimate the prevalence of multimorbidity in the Brazilian working population (≥18 years) and identify associated factors based on data from the 2013 national health survey (Pesquisa Nacional de Saúde).

**Methods:**

This was a cross sectional study based on data from the 2013 national health survey, which included n = 47,629 people aged 18 years or older. As part of the survey, participants were asked whether they had ever been diagnosed with any of several chronic diseases. The prevalence of multimorbidity in this population and its association with socioeconomic, lifestyle and occupational characteristics were examined. Bivariate analyses were used to calculate prevalence ratios and 95% confidence intervals. Multivariate analyses were conducted using Poisson regression and Wald’s tests to estimate the coefficients of significant variables.

**Results:**

The prevalence of multimorbidity was 19.98% (95% confidence interval: 19.29%-20.70%). Higher rates of multimorbidity were associated with female gender, age 60 years or older, living with a spouse, past history of smoking, low education levels (illiterate/primary), living in urban areas, having medical or dental insurance and a history of work accidents.

**Conclusions:**

The prevalence of multimorbidity in the Brazilian population is low. When present, multimorbidity is associated with specific occupational, socioeconomic and lifestyle characteristics.

## INTRODUCTION

Approximately 80% of appointments with primary care physicians are made by patients with multiple health conditions, or multimorbidity. The presence of multimorbidity is associated with increased complexity of care due to the need to treat two or more chronic conditions simultaneously. This issue is especially prevalent in older adults.^1^

The socioeconomic characteristics of an individual can either increase or decrease the risk of multimorbidity, and studies show that a lower socioeconomic status is associated with greater hardship and lower quality of life in daily activities.^2^ The existence of diverse social, economic and work conditions can be considered an important risk factor for health. The interference of these issues on the health-disease process either at an individual or collective level can be inferred from the increase in episodes of absence and illness in workers.^3^

Given the multitude of ways in which the health of individuals can be affected by their work, its importance as a determinant of population health and living conditions has considerably increased, prompting the Brazilian health care system (Sistema Único de Saúde; SUS) to take up the responsibility for programs and services targeting work and workers.^4^ The National Worker Health Policy of the Ministry of Health has been in effect since 2004, only 16 years after the promulgation of the Constitution, in 1988; subsequently, in 2012, the National Worker Health Policy was instituted by Ordinance No. 1,823. The aim of this policy was to reduce work-related accidents, injuries and illnesses by implementing health promotion, rehabilitation and surveillance programs in health care.^5^

According to the World Health Organization,^6^ current and future trends point to a shift in worker health profiles, characterized by increased life expectancy, older age, a decrease in chronic communicable diseases and increase in non-communicable chronic illnesses. Emerging threats to the health of workers also include issues such as increased job demands and diversity, precarious support systems and social protection, decreased autonomy, environmental degradation and worsening quality of life.^4^ Additionally, the health care system seems largely unprepared to meet the growing demands of individuals with non-communicable chronic illnesses.^7^

Given the impairments associated with multimorbidity and its known association with aging,^8^ it must be addressed as a public health issue, as the current and future risks of lifetime exposure to occupational risk factors include reduced productivity, work absence due to illness, and a higher risk of unemployment.^9^ While the literature on multimorbidity has grown at an unprecedented rate in recent years, few population-based studies have examined the issue.^10^ There has also been growing interest in the association between multimorbidity and worker health.

The aim of this study is to estimate the prevalence of multimorbidity and related factors in the Brazilian working population (≥18 years), in order to support and encourage strategic, political and social initiatives targeting worker health. We also aimed to contribute to the development of proposals and protocols for workers with multimorbidity in Brazil, which could provide effective and accessible resources to promote and protect the health of both formal and informal workers.

## METHOD

This was a cross-sectional study based on secondary data from the latest National Health Survey (Pesquisa Nacional de Saúde; PNS), a representative study of the adult Brazilian population (≥18 years of age) conducted by the Brazilian Institute of Geography and Statistics (Instituto Brasileiro de Geografia e Estatística; IBGE) and the Ministry of Health in 2013. The PNS was a home-based survey which aimed to investigate and describe the Brazilian population with regards to health status, lifestyle, prevalence of chronic illnesses, risk factors, as well as health care access and utilization.^11^

### POPULATION, SAMPLE AND LOCATION

The survey targets adults (≥18 years of age) living in private households throughout the country, with the exception of specially designated or sparsely populated areas, such as indigenous villages, army barracks, military bases, temporary lodgings, campsites, boats, prisons, penal colonies, jails, nursing homes, orphanages, convents and hospitals, as well as census sectors in indigenous territories.^12^

The sample was stratified in three stages: census tract (primary sampling units or collection units); household sectors; and adult residents (≥18 years). At each stage, participants were selected by simple random sampling.^11^

Sample size was based on the 2008 National Household Survey (Pesquisa Nacional por Amostra de Domicílios; PNAD) and the 2010 Population Census, which provided a sample size of 1,800 households per federal unit. According to Affonso,^13^ “federal units are a form of territorial organization of power, a method of articulation of a central power and regional and local authorities” (p. 321). Calculations based on the previously mentioned data estimated a total sample of 81,357 households. One individual was then recruited from each of these locations. Although a total of n = 64,308 individuals (≥18 years of age) were initially selected, 1,717 refused to participate and 2,389 could not be located, resulting in a final sample of n = 60,202, yielding a response rate of 86%.^11^ Only participants who were employed at the time of the survey (n = 47,629) were included in the present study.

The survey is divided into modules which contain a total of 743 questions about the household and the individual respondent.^12^ Interviewers were trained to conduct the survey using handheld computers to enter the data collected.^12^

The dependent variable was multimorbidity, defined as the presence of two or more noncommunicable chronic diseases in the same individual.^14^ The presence of the following conditions was examined during the survey: hypertension; diabetes; high cholesterol; heart conditions such as myocardial infarction, angina or heart failure; cerebrovascular accident or stroke; pulmonary disease or asthma (or asthmatic bronchitis); arthritis or rheumatism; chronic spinal cord injury, such as chronic back or neck pain, repetitive strain injury/work-related osteomuscular disorders, low back or sciatic pain, vertebral or disc problems; mental illnesses such as depression, schizophrenia, bipolar disorder, psychosis or obsessive-compulsive disorder (OCD); chronic kidney failure; or cancer.

The present study also analyzed socioeconomic, lifestyle and occupational characteristics, some of which were obtained from a combination of variables in the PNS survey. The variables analyzed were as follows: *Age group:* 18-24 years; 25-39 years; 40-59 years; or 60 years or older. *Education level*^15^: illiterate; primary (complete primary and incomplete secondary education); secondary (complete secondary and incomplete university education); and higher education (complete higher education). *Married*: Yes/No. *Residential location:* urban or rural. *Health insurance:* Does the respondent have health insurance: Yes/No. *Smoking status:* nonsmoker; former smoker; current smoker. *Alcohol intake:* no alcohol intake; moderate alcohol intake; excessive alcohol intake. *Work environment:* Indoors; outdoors; both. *Occupational accidents:* Was the respondent involved in an occupational accident in the past 12 months, regardless of occupation: Yes/No. *Accident-related injuries:* Yes/No. *Night work:* Yes/No. *Shift work:* Yes/No. *Hours worked:* the only quantitative variable in the study.

Statistical methods were used to analyze the prevalence of multimorbidity and its association with socioeconomic, lifestyle and occupational characteristics in the population studied. Bivariate analyses were used to calculate prevalence ratios (PR) and 95% confidence intervals (95%CI). Multivariate analyses were conducted using Poisson regression with robust variance estimation. Analyses were performed using Stata, version 14 (StataCorp LLC, Texas, USA).

The PNS was originally approved by the National Research Ethics Committee (Comissão Nacional de Ética em Pesquisa; CONEP) on July 8th, 2013, under protocol number 10853812.7.0000.0008. The present study was based on secondary data from the PNS collected from the official website of the Brazilian Ministry of Health, and does not require ethical approval, as outlined in the National Health Council Resolution 466/2012.

## RESULTS

The overall prevalence of multimorbidity in the sample was 19.89% (95%CI, 19.29%-20.70%). Descriptive analyses showed that prevalence rates were highest in individuals aged 60 years or older (PR = 46.71%; 95%CI, 44.36%-49.07%) and in former smokers (PR = 32.41%; 95%CI, 30.52%-34.35%). Multimorbidity was also associated with accident-related injuries (PR = 32.68%; 95%CI, 23.23%-43.78%), illiteracy (PR = 26.15%; 95%CI, 25.01%-27.31%), female gender (PR = 24.91%; 95%CI, 23.90%-25.95%) and a history of occupational accidents (PR = 24.71%; 95%CI, 20.58%-29.38%). The prevalence of multimorbidity was also slightly higher in the following groups: people who lived with a partner; non-drinkers; residents of urban areas; people with medical or dental insurance; non-night workers; indoor workers; and shift workers (Table 1).

**Table 1:** Prevalence (%) of multimorbidity in the Brazilian working population according to socioeconomic and health characteristics assessed by the 2013 National Health Survey (*Pesquisa Nacional de Saúde*) (n = 47,629).

Variables	P (%)	95%CI	
		LL	UL
Multimorbidity			
No	80.02%	79.3%	80.71%
Yes	19.98%	19.2%	20.70%
Gender			
Male	15.46%	14.6%	16.31%
Female	24.91%	23.9%	25.95%
Age group			
18-24	4.64%	3.66%	5.87%
25-39	10.54%	9.72%	11.42%
40-59	27.80%	26.5%	29.05%
60+	46.71%	44.3%	49.07%
Married			
No	16.79%	15.8%	17.80%
Yes	21.92%	21.0%	22.83%
Alcohol intake			
None	21.41%	20.6%	22.25%
Moderate	16.77%	15.3%	18.28%
Excessive	14.50%	12.6%	16.58%
Smoking status			
Nonsmoker	16.90%	16.1%	17.68%
Current smoker	20.69%	19.0%	22.47%
Former smoker	32.41%	30.5%	34.35%
Education level			
Illiterate	26.15%	25.0%	27.31%
Secondary	14.38%	13.4%	15.35%
Higher Education	17.50%	16%	19.11%
Residential location			
Urban	20.36%	19.5%	21.15%
Rural	17.51%	16.1%	19.02%
Medical or dental insurance			
No	18.85%	18.0%	19.66%
Yes	22.50%	21.1%	23.91%
Occupational accidents			
No	19.85%	19.1%	20.57%
Yes	24.71%	20.5%	29.38%
Night work			
No	17.33%	16.5%	18.18%
Yes	17.28%	15.5%	19.11%
Work environment			
Indoors	17.50%	16.4%	18.58%
Outdoors	17.15%	15.8%	18.53%
Both	17.12%	15.6%	18.72%
Accident-related injury			
No	19.92%	19.2%	20.63%
Yes	32.68%	23.2%	43.78%
Shift work			
No	17.29%	16.5%	18.08%
Yes	19.21%	14.5%	24.95%

95%CI: 95% confidence interval; LL: lower limit; UL: upper limit; P: prevalence.

Table 2 shows the bivariate analyses of the association between multimorbidity and socioeconomic, health and occupational characteristics. The results showed that former smokers were twice as likely to have multimorbidity than non-smokers or current smokers. Other factors associated with multimorbidity include work-related injury (PR = 1.64; 95%CI, 1.19%-2.25%), low education level (illiterate/primary) (PR = 1.49; 95%CI, 1.35%-1.64%), living with a partner (PR = 1.30; 95%CI, 1.21%-1.39%) and a history of occupational accidents (PR = 1.24; 95%CI, 1.03%-1.49%). Variables such as male gender, being 18-24 years of age, having no medical or dental insurance, excessive alcohol intake, completing secondary education and living in rural areas were negatively associated with multimorbidity. Bivariate analysis showed that night work, the type of occupational environment and shift work were not significantly associated with the prevalence of multimorbidity.

**Table 2:** Bivariate analysis of factors related to multimorbidity in the Brazilian working population, according to the 2013 National Health Survey (*Pesquisa Nacional de Saúde*) (n = 47,629).

Variables	PR	95%CI		p value
		LL	UL	
Gender				
Male	0.62	0.58%	0.66%	<0.01
Female	1			
Age group				
18-24	0.09	0.07%	0.12%	<0.01
25-39	0.22	0.20%	0.24%	<0.01
40-59	0.59	0.55%	0.63%	<0.01
60+	1			
Married				
No	1			
Yes	1.30	1.21%	1.39%	<0.01
Medical or dental insurance				
No	0.83	0.77%	0.90%	<0.01
Yes	1			
Alcohol intake				
None	1			
Moderate	0.78	0.71%	0.85%	<0.01
Excessive	0.67	0.58%	0.77%	<0.01
Smoking status				
Non-smoker	1			
Smoking	1.22	1.11%	1.34%	<0.01
Former smoker	1.91	1.78%	2.05%	<0.01
Education level				
Illiterate/primary	1.49	1.35%	1.64%	<0.01
Secondary	0.82	0.73%	0.91%	<0.01
Higher education	1			
Residential location				
Urban	1			
Rural	0.86	0.78%	0.94%	<0.01
Workload				
Hours	0.99	0.98%	0.99%	<0.01
Occupational accidents				
No	1			
Yes	1.24	1.03%	1.49%	<0.01
Night work				
No	1			
Yes	0.99	0.89%	1.11%	0.95
Work environment				
Outdoors	1			
Indoors	1.02	0.92%	1.12%	0.87
Both	0.99	0.88%	1.12%	0.87
Work-related injury				
No	1			
Yes	1.64	1.19%	2.25%	<0.01
Shift work				
No	1			
Yes	1.11	0.84%	1.46%	0.45

95%CI: 95% confidence interval; LL: lower limit; UL: upper limit; p value: probability of significance; PR: prevalence ratio.

The multivariate analyses, shown in Table 3, revealed that multimorbidity is significantly associated with the following variables: female gender (PR = 1.72; 95%CI, 1.59%-1.86%), age 60 years or older (PR = 6.93; 95%CI, 5.35%-8.97%), a history of occupational accidents (PR = 1.50; 95%CI, 1.25%-1.79%), living with a partner, and being illiterate or having only primary education.

**Table 3:** Multivariate-adjusted prevalence ratios (PRadj) for multimorbidity in the Brazilian working population, based on the 2013 National Health Survey (*Pesquisa Nacional de Saúde*) (n = 36,442).

Variables	PRadj	95%CI		p value
		LL	UL	
Gender				
Male	1			
Female	1.72	1.59%	1.86%	<0.01
Age group				
18-24	1			
25-39	1.80	1.41%	2.30%	<0.01
40-59	4.23	3.33%	5.39%	<0.01
60+	6.93	5.35%	8.97%	<0.01
Married				
No	1			
Yes	1.20	1.11%	1.31%	<0.01
Medical or dental insurance				
No	1			
Yes	1.23	1.12%	1.34%	<0.01
Education level				
Illiterate/primary	1.12	1.00%	1.26%	<0.01
Secondary	0.96	0.86%	1.08%	<0.01
Higher education	1			
Smoking status				
Nonsmoker	1			
Smoking	1.19	1.06%	1.33%	<0.01
Former smoker	1.51	1.38%	1.65%	<0.01
Residential location				
Urban	1.13	1.01%	1.26%	<0.01
Rural	1			
Occupational accidents				
No	1			
Yes	1.50	1.25%	1.79%	<0.01
Workload				
Hours	0.99	0.99%	1.00%	<0.01

95%CI: confidence interval; LL: lower limit; UL: upper limit; p value: probability of significance; PRadj: adjusted prevalence ratios.

## DISCUSSION

The present findings revealed an overall prevalence of 19.98% for multimorbidity in the Brazilian workforce. This figure is lower than that observed in other populations. Excoffier et al.,^16^ for instance, conducted a study with 118 general practitioners in a sentinel network, each of whom collected data from 25 patients treated over a period of 2 weeks, revealing a prevalence of 52.1% for multimorbidity. In another study, conducted by Prazeres and Santiago,^17^ a random sample of general practitioners working in the National Health Service in Portugal recruited a total of 1,500 patients, 57.2% of whom had two or more chronic illnesses. The study in question screened for 147 chronic conditions.

The low prevalence rate in the present study may be due to a lack of knowledge or orientation to attend to signs and symptoms of disease onset or progression, as well as a lack of access to health care professionals and services.^18^ In the absence of health screening or monitoring, there can be no diagnosis or effective treatments. The fact that the present study relied on self-reported diagnoses may have also influenced our findings.

The prevalence of multimorbidity was higher in individuals who reported occupational accidents than in those without such a history. Although the present findings support an association between multimorbidity and work accidents, longitudinal studies are still required to test for causal relationships between these variables.

The number of reported occupational accidents increases every year. An estimated 317 million such incidents occur every year worldwide.^19^ The International Labor Organization (ILO) also notes that 2.3 million workers every year die from work-related activities.^20^

According to the latest report from the 4th National Conference on Worker Health, a total of 717,911 work accidents were recorded in Brazil in 2013, representing a major cause of work absence in the country.^21^ The fact that approximately 18.7 million Brazilians work in the informal sector and have no access to social welfare or labor protections^22^ may also contribute to underreporting regarding the safety and health of Brazilian workers.

The increasing rate of underreporting contributes to the growing distance between official records and real-world data. Based on data collected by the IBGE in 2013, Gonçalves et al.^19^ estimated that, for every accident notified or reported to social welfare services in Brazil, seven go unreported. It is therefore possible that the actual prevalence of these incidents and their association with other variables are higher than observed in the present study.

People aged 60 years or older had a higher prevalence of multimorbidity than those aged 18 to 24 years. The literature suggests that the frequency of multimorbidity tends to increase proportionately with age.^8^ A study of residents in all 27 capital cities of Brazil in 2013 revealed that individuals aged 50-59 were up to 30 times more likely than those aged 18 to 29 to have a chronic illness; in other words, older age was associated with a higher risk of multimorbidity.^23^

Female gender was also strongly associated with multimorbidity in the workforce, a finding that is well established in the literature, and may be related to the longer life expectancy of women relative to men. Women tend to use health care services more often than their male counterparts, and as such, are more likely to receive a medical diagnosis.^24^ Studies have also identified several genetic, occupational, lifestyle and biopsychosocial factors that increase susceptibility to multimorbidity in women.^25^

Married individuals also had a higher likelihood of multimorbidity than their single counterparts. Yet a study conducted in the rural region of Matlab, Bangladesh, found that multimorbidity rates were higher among single individuals.^25^

Those with no medical or dental insurance also had a higher prevalence of multimorbidity. It is possible that these individuals come from less privileged backgrounds and have no access to private health care, which offers better quality service, especially with regards to communication and interactions between patients and health care professionals.^26^ As a result, individuals with greater access to private health care may be more likely to receive a diagnosis than those with no access to these resources. Therefore, some of the differences in multimorbidity rates may not be attributable to actual discrepancies in illness rates, but to access to health care providers that can diagnose conditions that are already present.

The analysis of education levels revealed that people with no formal education or only primary schooling have higher rates of multimorbidity than the remainder of the sample. It is possible that these individuals have less access to information and health care, including basic knowledge regarding protection and prevention of risk factors, as well as methods of health promotion.^27^

A study conducted in the city of Florianópolis in southern Brazil identified some key differences between individuals with low vs high rates of multimorbidity. The lowest rates were usually observed in younger and more educated individuals, who are likely to have better access to information and preventive health knowledge. A high prevalence of multimorbidity, on the other hand, was associated with older age, lower socioeconomic status (class C), less than eight years of education and obesity,^28^ all of which may contribute to lower access to information and greater exposure to health risks.

Former smokers also had higher rates of multimorbidity than nonsmokers and current smokers. Multimorbidity is a multifactorial condition, and smoking is considered a major preventable cause of chronic noncommunicable disease.^23^ Studies suggest that the early aging and increased incidence of illnesses in smokers can be explained by the physicochemical processes prompted by substances found in cigarettes, which result in oxidative stress and cellular damage.^27^

Living in urban areas was also associated with increased rates of multimorbidity. This may be attributable to the easier access to health care in urban regions, which may lead to more frequent medical appointments and hospitalizations. The association between multimorbidity and urban living may also be affected by the impact and prevalence of unhealthy lifestyle habits, rapid urbanization and to poorly managed and exclusive globalization.^8^

The cross-sectional nature of the present study prevented the identification of causal relationships between variables, since the direction of causality cannot be determined when risk factors and outcomes are assessed simultaneously. This constitutes a limitation of the present study. A second potential limitation is that, although the present study was based on representative population data, diagnoses were determined by selfreport. As a result, findings may have been influenced by comprehension issues, recall bias and under- or overestimated prevalence rates. However, the sample size and methodological rigor add to the robustness of the present findings. Since this was a populationbased study, it also contributes to the identification of outcomes and risk factors across the country.

## CONCLUSIONS

This study revealed a low prevalence of multimorbidity in Brazilian workers, which may reflect a difficulty in capturing the multidimensional nature of multimorbidity.

Future population-based studies in Brazil, such as the PNS, should collect additional information on multimorbidity, its prevalence and impact on workers, in order to address the limitations identified in the present study and provide a more accurate picture of the Brazilian reality. There is also a need for longitudinal population studies that provide a more detailed analysis of the association between multimorbidity and work-related problems, which could not be assessed in the present study, but remain a topic of interest in the international literature on worker health.^29^

Nevertheless, the present study makes an important contribution to the literature, revealing some of the factors that influence multimorbidity and providing information to support and enable the development of strategic, political and surveillance initiatives to promote worker health. This study also encourages continued discussion on worker health and illness in Brazil.
